# Relationship between body size and sexual size dimorphism in syringophilid quill mites

**DOI:** 10.1007/s00436-022-07437-3

**Published:** 2022-01-24

**Authors:** Lajos Rózsa, Evelyn Moldovan

**Affiliations:** 1 Institute of Evolution, ELKH Centre for Ecological Research, Konkoly-Thege street 29-33, Budapest, H-1121 Hungary; 2 Hungarian Department of Biology and Ecology, Babeș-Bolyai University, Cluj-Napoca, Romania

**Keywords:** Quill mites, Sexual selection in parasites, Rensch’s rule, Converse Rensch’s rule

## Abstract

A positive relationship of body size and sexual size dimorphism (males’ size relative to females), called Rensch’s rule, is often observed in comparisons within non-parasitic taxa. However, this allometric relationship has rarely been tested in comparisons across closely related parasite species. Since male sexual rivalry is often regarded as the main cause of this phenomenon, the present study tests this rule in a taxon where sexual selection is almost totally absent in males. Body size data of (non-physogastric) female and male quill mites (Acari: Syringophilidae) were gathered from the literature to investigate this relationship. The data set consisted of 113 species representing 8 genera. For the data set as a whole, increasing body size came together with decreasing relative body size of males (relative to females), a phenomenon known as converse Rensch’s rule. Repeating the same analysis for the 8 genera separately, similar patterns were found in 4 significant and 3 non-significant cases. There was a significant tendency to comply with Rensch’s rule only in one genus, the *Neoaulonastus*. Thus, converse Rensch’s rule is the primary trend in syringophilid quill mites that appears repeatedly and independently in several genera. This phenomenon is probably caused by their extreme inbreeding, which strongly reduces sexual competition among males in this taxon.

## Introduction

Most parasites reproduce sexually, or at least both sexually and asexually, giving rise to sexual selection as a potentially influential force on their evolution. However, sexual selection is not easy to measure directly. Most often only its indirect effects are detected, such as the sexually selected morphological traits of parasites. Sexual dimorphism in body size is widespread among dioecious parasites, such as in nematodes, acanthocephalans, gamasid mites, fleas, and lice (see, e.g., Poulin 1997; Caddigan et al. 2017; Surkova et al. 2018; Piross et al. 2019). It is of outstanding importance since body size influences metabolism, length of development, longevity, sexual competitive ability, and fecundity (for insects, see, e.g., Honěk 1993; Waller and Svensson 2017; Beukeboom 2018). Female fecundity likely increases with body size in ectoparasitic arthropods, because larger females can produce more offspring (Villa et al. 2018). Similarly, larger males can produce more sperm than smaller ones, which is an adaptive advantage under the circumstances of sperm competition that characterizes male-male rivalry in many ectoparasitic arthropods (Pap et al. 2013; Rózsa et al. 2015). Moreover, body size data are readily available from the taxonomic literature, such as species descriptions (for ectoparasites, see, e.g., Harnos et al. 2017).

In comparisons across closely related species, male body size relative to female size tends to increase with the average size of the species, an allometric relationship called Rensch’s rule (RR). More specifically, RR postulates that sexual size dimorphism decreases with the species body size in taxa where males are smaller than females, and it increases in taxa where males are larger than females (Rensch 1959). Several free-living (i.e., non-parasitic) animals, and most often vertebrates, have been analyzed in this respect, and a majority of them proved the validity of RR (see, e.g., Smith and Cheverud 2002; Székely et al. 2004). Nonetheless, opposite trends (where male relative size decreases with the size of the species) also occur in some taxa, a phenomenon known as converse Rensch’s rule (ConRR). This trend characterizes, among others, several bird families (Webb and Freckleton 2007).

Although parasitism is a most widespread life strategy in the biosphere (Poulin and Morand 2014), almost all studies on RR or ConRR focus on free-living animals, with very few studies on parasites. Poulin (1996) showed that parasitic copepods follow RR, but parasitic nematodes do not comply (Poulin 1997). Surkova et al. (2018) found that fleas (Siphonaptera) obey, but parasitic gamasid mites disobey RR. Piross et al. (2019) recently showed that two families of avian lice (Menoponidae, Philopteridae) comply with RR, and the third one (Ricinidae) complies with ConRR. The potential reasons for these contradictory patterns are not clearly understood. Since sexual selection (and male-to-male sexual rivalry in particular) is often argued to be the driving force of RR (see, e.g., Székely et al. 2004), it is worth testing its validity in sexually reproducing taxa that are characterized by the absence of sexual selection. RR is predicted not to apply to these taxa.

Syringophilid quill mites constitute a species-rich (406 species in 63 genera up to the present, Zmudzinski et al. 2020) family of prostigmatic mites (Acari: Acariformes: Prostigmata) that are permanently associated with avian (Vertebrata: Aves) feathers. They likely appeared about 180–185 million years ago in the Early Jurassic, presumably on feathered dinosaurs (Dabert et al. 2010). All known representatives of the family inhabit feather quills. Members of the subfamily Syringophilinae tend to inhabit the quills of primaries, secondaries, and wing coverts, while Picobiinae species are found in the quills of body contour feathers (Skoracki et al. 2012a).

They exhibit a remarkable life cycle. A single fertilized female enters the soft calamus of a developing feather through the superior umbilicus (Casto 1974). This opening is getting closed soon, and the female will produce offspring, most often a single male and several females, developing in this enclosed space. The offspring then fertilize each other and produce one more generation still enclosed in the same feather quill. Again, only a single male offspring is produced by each female, which will fertilize their sisters and cousins. Finally, only fertilized females disperse to search for developing feathers either on the same host individual or on another one (Kethley 1971; Skoracki et al. 2012a). The most frequent type of transmission is probably the parent-offspring route.

Given that brothers, sisters, and first cousins tend to fertilize each other, the population structure of quill mites must be highly inbred. On some rare occasions, however, two or more females may invade the same quill to found new subpopulations in parallel (see, e.g., Casto 1974; Skoracki et al. 2020), potentially giving rise to sexual competition and sexual selection between the males. Nevertheless, such events must be rare because of the space limitation within the feather quills. Given that sexual selection is almost totally lacking in this taxon, the objective of this study was to test the predicted absence of RR in quill mites.

## Materials and methods

Different genera that inhabit different microhabitats in the bird plumage were included. Thus, as an arbitrary decision, 9 relatively species-rich genera were chosen for the present analysis: *Syringophiloidus*, *Syringophilopsis*, *Aulobia*, and *Torotrogla* which infest the secondaries and primaries, *Aulonastus* and *Neoaulonastus* which infest the small coverts, and *Picobia*, *Gunabopicobia*, and *Neopicobia* which infest the contour feathers.

The taxonomic literature was not sampled to gather data; instead, every species description ever published was involved. The comprehensive list of species descriptions by Zmudzinski et al. (2020) was used as a starting point for orientation in the literature. The body size data were obtained from the species descriptions of Bochkov (2001), Bochkov and Apanaskevich (2001), Bochkov and Galloway (2001, 2004), Bochkov and Mironov (1998, 1999), Bochkov et al. (2000, 2001, 2004, 2009), Chirov and Kravtsova (1995), Fain et al. (2000), Glowska (2014), Glowska and Skoracki (2011), Glowska et al. (2015, 2018), Kethley (1970), Klimovičová et al. (2016), Nattress and Skoracki (2007), Sikora et al. (2011, 2014, 2016), Skoracki (1999, 2002a, b, 2004a, b, c, 2011, 2017), Skoracki and Bochkov (2010), Skoracki and Dabert (1999, 2000, 2001a, b, 2002), Skoracki and Glowska (2008a, b), Skoracki and Hromada (2013), Skoracki and Magowski (2001), Skoracki and Mironov (2013), Skoracki and O’Connor (2010), Skoracki and Sikora (2003, 2014), and Skoracki et al. (2000b, 2001a, b, 2002, 2003, 2004, 2008a, b, 2010a, b, c, d, 2011, 2012b, 2013a, b, 2014a, b, c, 2016a, b, c, 2017, 2018).

In certain arthropods, there is an alternative female phenotype characterized by a greatly enlarged abdomen (or hysterosoma, in mites) size. These females, called physogastric females, occur together with “normal” females. This phenomenon is well known in the quill mite subfamily Picobiinae but not in Syringophilinae (Skoracki et al. 2012a). Thus, the adult females of the former subfamily may have two alternative phenotypes: the more frequent non-physogastric and the less common physogastric form. The adaptive value of this dimorphism is unknown (Skoracki et al. 2000a). To ensure comparability of body size data across the two subfamilies, all physogastric body size data were excluded from the present study.

The majority of species were excluded from the present analyses because either the male or (in rare occasions) the non-physogastric female body length was not known. The genus *Gunabopicobia* was excluded because it had only a single species where both body length data were available. Further, *Torotrogla calcarius* Skoracki 2004a, b, c exhibited an unusual characteristic in the sense that the males were larger than the single female known. The author of this species description stated that this female specimen was slightly distorted and its length measurement was likely incorrect (Maciej Skoracki, personal communication); thus, it was also excluded from the data set. Worth noting that, if included, this data point would further increase the statistical significance of results.

The total body length (in micrometers) values were used as a measure of body size. Whenever it was possible, the data of the holotype specimens were used. Otherwise, the mean of the extreme values of the body length range of the series of paratype specimens was calculated. Males are always smaller-sized than females in this taxon. In the present sample of species, female body length ranged between 405 and 1320, and male body length between 270 and 955 μm.

Raw data of body length did not vary more than one magnitude and, therefore, logarithmic (or any other) transformation of data was not applied. Following Reiczigel et al. (2014), homoscedasticity of data was tested using the *F*-test, and normality of the residuals was checked using the Shapiro-Wilk test (*p* > 0.05 in all cases). Reduced major axis (RMA) regressions (Legendre 2018) were used to describe how male and female body lengths were correlated. A slope of the regression line >1 was considered as a proof of RR, and a slope <1 as a proof of ConRR. The significance levels were judged using the 95% of confidence intervals of the slopes.

Closely related species are more likely to share similar characters than distantly related ones. Therefore, when analyzing statistical covariation between two characters across a set of species, it is necessary to control for phylogenetic associations (Felsenstein 1985). In the present case, unfortunately, the phylogeny of syringophilids is not adequately known to apply advanced statistical methods for this purpose. Therefore, this goal was approached by the following simplified way. First, the analysis was carried out using the data of all species regardless of their taxonomic position. Second, the same calculations were carried out for all the genera separately to see if the same pattern is repeated independently. Presuming that the taxonomic classification mirrors the true phylogeny of species, this is regarded as a simplified statistical control for phylogeny.

## Results

**Fig. 1:**
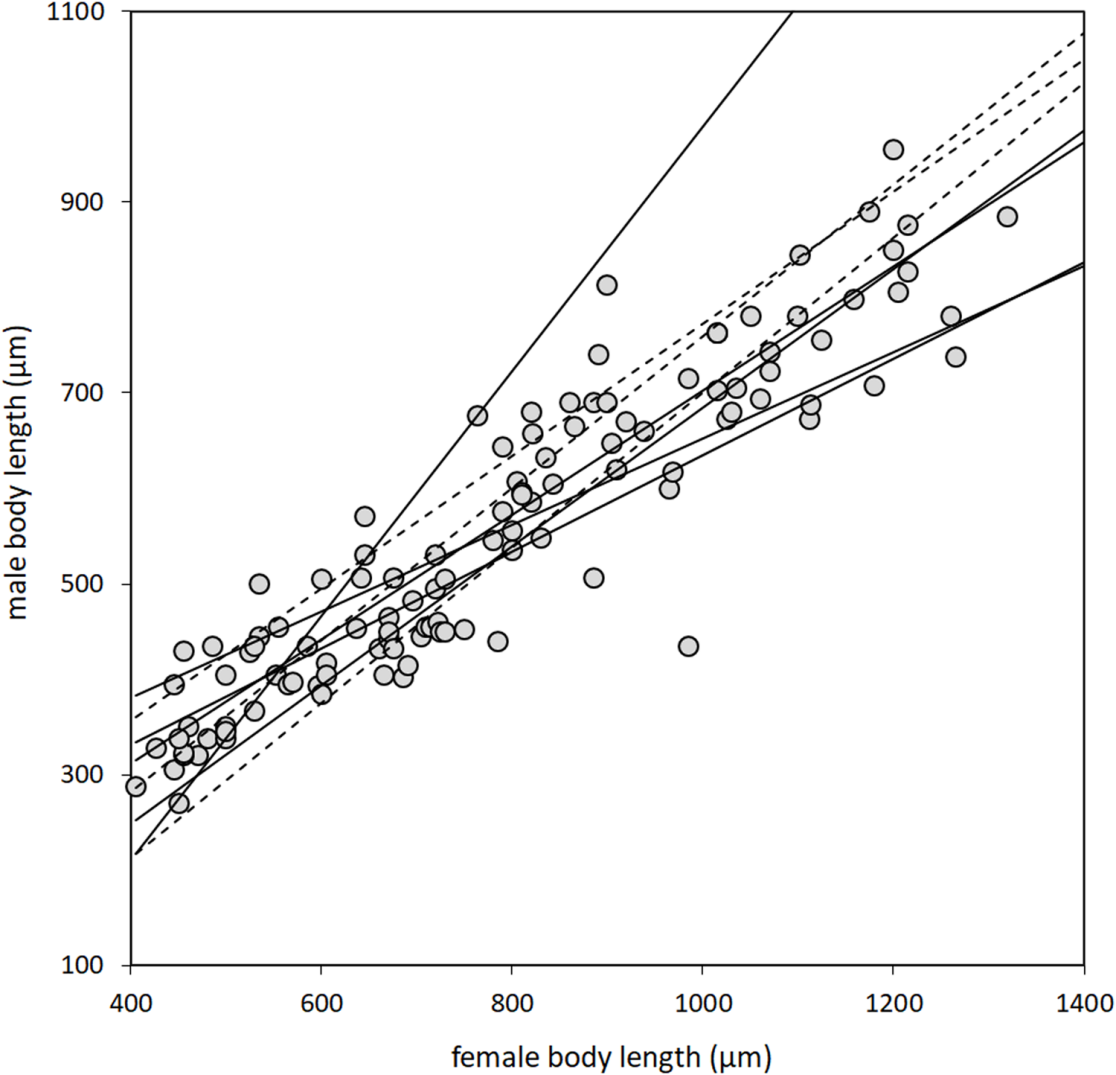
Male body length as a function of female body length in 113 species of syringophilid quill mites. Each dot indicates a species, and the different genera are not differentiated. RMA regression lines of the eight genera are indicated separately. Solid lines signify those that differ from slope = 1 significantly (*p* < 0.05). Their slopes are < 1 indicating that they comply ConRR, with the exception of *Neoaulonastus* which complies RR (slope > 1). Dashed lines signify genera that comply ConRR non-significantly (*p* > 0.05)

The data set contained a total of 113 species belonging to 8 genera that inhabit 3 different microhabitats in the host plumage. As a whole, the data followed ConRR in the sense that the entire 95% confidence interval of its slope fell within the 0 < slope < 1 range. Considering the slopes of the 8 genera separately yielded in roughly similar patterns. Only one genus, namely, *Neoaulonastus*, exhibited a significant trend to follow RR (slope > 1). Worth noting that this genus also had the lowest sample size (number of species = 6). Independently from each other, all the other genera followed ConRR, and this trend was significant in 4 and non-significant in 3 cases (Table 1, Fig. 1).

**Table 1:** Reduced major axis regression models for the relationship between female body length and male body length for the whole family of quill mites and for 8 genera separately. Slopes < 1 indicate agreement with ConRR, while slopes > 1 indicate agreement with RR

Taxon	*N* species	*R*^2^	Slope	95% CI lower	95% CI upper	Trend	Significance 95%
Syringophilidae	113	0.851	0.689	0.640	0.741	ConRR	S
*Aulonastus*	8	0.470	0.795	0.405	1.560	ConRR	NS
*Torotrogla*	11	0.416	0.693	0.400	1.199	ConRR	NS
*Neoaulonastus*	6	0.971	1.281	1.014	1.617	RR	S
*Aulobia*	10	0.606	0.811	0.496	1.326	ConRR	NS
*Neopicobia*	7	0.768	0.452	0.266	0.766	ConRR	S
*Picobia*	14	0.373	0.504	0.312	0.814	ConRR	S
*Syringophiloidus*	25	0.524	0.725	0.541	0.973	ConRR	S
*Syringophilopsis*	32	0.505	0.651	0.502	0.844	ConRR	S

The eight slopes of the genus-level RMA regression models had a mean of 0.739 that differs significantly (one-sample *t*-test, *t* = 2.914, df = 7, *p* = 0.022) from the hypothetical value of 1 (which would signify no relationship between body size and sexual size dimorphism).

## Discussion

As demonstrated above, ConRR characterizes the family quill mites (Syringophilidae) as a whole. It is not a phylogenetic artifact, rather a phenomenon that appears repeatedly and independently across several, if not all, quill mite genera. Published interpretations of RR and ConRR are diverse and often contradicting, as summarized by Piross et al. (2019). One possible explanation of the present results is outlined below.

Increasing body size is likely to come with increasing costs and benefits for females. First, larger females evidently occupy more space and consume more food to develop and maintain their bodies, decreasing the space and other resources available for their own siblings and offspring. Due to their increased metabolism, presumably, larger bodies evoke more intensive host defenses, such as more intense immune response or more intense preening and grooming while the mite is out of the quill. These factors likely constitute increased costs of larger body sizes. On the other hand, larger females likely enjoy a reproductive benefit; they can probably produce a greater number and larger-sized offspring (like in feather lice, see Villa et al. 2018). In male individuals, the potential costs of larger body size are similar to that of females; more space occupied, more food consumed, and more defensive responses evoked. The potential benefits of larger male body size, however, are (almost) absent. Given that sexual rivalry is almost totally absent in males, larger size does not ensure a reproductive advantage in the male sex. This is a possible reason why the increase of female body size is not followed by a comparable increase of male body size in syringophilid quill mites. This interpretation corresponds the so-called sexual selection hypothesis of RR (Fairbairn and Preziosi 1994; Fairbairn 1997).

If this explanation is correct, it may also support the recent results and interpretations of Piross et al.’s (2019) study on avian lice (Phthiraptera). Among avian lice, only one family, the Ricinidae, complied with ConRR. Note that this family possesses the largest body size relative to the host body size (Harnos et al. 2017). Therefore, only a few individuals may occur on an individual host. Moreover, their prevalence also tends to be low (Rheinwald 1968; Nelson 1972), and thus, multiple infestations are necessarily rare. These factors make it likely that inbreeding is high in ricinid lice, a presumption also supported by the relatively low sex-ratio (proportion of males) in this family.

Briefly, the present results suggest that parasite taxa with pronounced inbreeding and, therefore, reduced male-male competition are predicted to comply with ConRR, rather than RR.

## Data Availability

Not applicable.
